# Oxidized low-density lipoprotein associates with cardiovascular disease by a vicious cycle of atherosclerosis and inflammation: A systematic review and meta-analysis

**DOI:** 10.3389/fcvm.2022.1023651

**Published:** 2023-01-16

**Authors:** Christin G. Hong, Elizabeth Florida, Haiou Li, Philip M. Parel, Nehal N. Mehta, Alexander V. Sorokin

**Affiliations:** Section of Inflammation and Cardiometabolic Diseases, National Heart, Lung, and Blood Institute, National Institutes of Health, Bethesda, MD, United States

**Keywords:** cardiovascular disease, atherosclerosis, inflammation, oxidized low-density lipoprotein, lipids

## Abstract

**Background:**

Low-density lipoprotein cholesterol (LDL-C) is an established marker for cardiovascular disease (CVD) and a therapeutic target. Oxidized LDL (oxLDL) is known to be associated with excessive inflammation and abnormal lipoprotein metabolism. Chronic inflammatory diseases confer an elevated risk of premature atherosclerosis and adverse cardiovascular events. Whether oxLDL may serve as a potential biomarker for CVD stratification in populations with chronic inflammatory conditions remains understudied.

**Objective:**

To perform a systematic review and meta-analysis evaluating the relationship between oxLDL and CVD (defined by incident CVD events, carotid intima-media thickness, presence of coronary plaque) in patients with chronic inflammatory diseases.

**Methods:**

A systematic literature search was performed using studies published between 2000 and 2022 from PubMed, Cochrane Library, Embase (Elsevier), CINHAL (EBSCOhost), Scopus (Elsevier), and Web of Science: Core Collection (Clarivate Analytics) databases on the relationship between oxLDL and cardiovascular risk on inflamed population. The pooled effect size was combined using the random effect model and publication bias was assessed if *P* < 0.05 for the Egger or Begg test along with the funnel plot test.

**Results:**

A total of three observational studies with 1,060 participants were ultimately included in the final meta-analysis. The results demonstrated that oxLDL is significantly increased in participants with CVD in the setting of chronic inflammatory conditions. This meta-analysis suggests that oxLDL may be a useful biomarker in risk stratifying cardiovascular disease in chronically inflamed patients.

## Introduction

Atherosclerosis is a complex pathophysiological process driven by metabolic derangements, lipid accumulation, and inflammation (1–3). While it may be clinically silent at early stages, atherosclerotic lesions often transform into vulnerable plaques prone to rupture and incite subsequent adverse events, including myocardial infarction, stroke and death (4, 5). Coronary artery disease (CAD) is an atherosclerotic cardiovascular disorder and continues to be the leading cause of mortality worldwide, despite advancements in treatments (6, 7). While traditional cardiovascular (CV) risk factors contribute to the pathogenesis of CAD, other novel risk factors may be involved. In particular, systemic inflammation has thought to play a role in the development and progression of CAD (1, 8). Growing body of evidence has shown that chronic inflammatory diseases, such as psoriasis (PSO), rheumatoid arthritis (RA), human immunodeficiency virus (HIV), and systemic lupus erythematosus (SLE) are associated with accelerated atherosclerosis and premature adverse CV events (9–14). In fact, such conditions are now considered independent risk factors for cardiovascular disease (CVD) (15). However, traditional CV risk stratification using Framingham risk score and age is suboptimal in assessing CVD risk in patients with chronic inflammatory conditions (16–18). For example, severe psoriasis has been shown to confer an additional 6.2% increase in long-term risk of CVD based on Framingham Risk score (19). Given the elevated CVD risk and current challenges in evaluating CVD in these inflamed populations, it is necessary to identify prognostic tools that will adequately capture and assess CVD risk.

Low-density lipoprotein cholesterol (LDL-C) is a known biomarker of cardiovascular disease (CVD) (20, 21). Pharmacological reduction of LDL-C is considered a main tool in the primary prevention of atherosclerotic cardiovascular disease (ASCVD); however, the issue of residual atherosclerotic risk that remains in patients with decreased LDL-C and elevated high density lipoprotein-cholesterol (HDL-C) is of additional clinical concern (22). Alternativly, other LDL-related lipoprotein species, such as small-dense LDL (sdLDL), lipoprotein (a) [Lp (a)], and oxLDL, have been shown to be reliable markers of CVD risk prognosis as well (23–25). Oxidative stress contributes to atherosclerotic plaque formation by stimulating activation of macrophages and vascular smooth muscle cells, increasing extracellular cholesterol accumulation within vessel walls, and transforming macrophages into pro-inflammatory and pro-thrombotic phenotypes (26). The observed critical step in atherosclerotic plaque build-up, the foam cell formation, is triggered by the uptake of oxLDL by macrophages through scavenger receptors, such as CD36, as well as lectin-like oxLDL receptor (LOX-1) (27–29). Previous studies have found that circulating oxLDL associates with every stage of atherosclerosis, from subclinical atherosclerosis to overt cardiovascular disease, including hypertension, coronary and peripheral arterial disease, acute coronary syndromes, and ischemic cerebral infarction, and has prognostic value in estimating CVD risk (25, 30, 31). Indeed, elevated levels of oxLDL were shown to predict myocardial infarction in the Health ABC cohort, even after adjusting for age, gender, race, smoking, and metabolic syndrome (30). OxLDL may even be associated with arterial aging, as a recent study found that oxLDL demonstrated predictive value of arterial stiffness, as measured by pulse-wave velocity, in patients with normal to mildly reduced renal function (32). Further, oxLDL is linked with metabolically dysfunctional pathologies frequently associated with CVD, including obesity, metabolic syndrome, and diabetes mellitus (25). Thus, oxLDL has recently become an important therapeutic target for CVD and has been recognized as a biomarker for CAD and other age-related atherosclerotic processes (24, 31, 33, 34). However, to what extent oxLDL contributes to CVD within systemic inflammation and whether it has any clinical utility in CVD risk stratification for such populations remain understudied. Therefore, we conducted a systematic review to examine the available evidence and aimed to investigate the association between oxLDL levels and CVD in the setting of chronic inflammation by meta-analysis.

## Methods

### Search strategy

The systematic review and meta-analysis were conducted according with the Preferred Reporting Items for Systematic reviews and Meta-Analyses guidelines (35) and the protocol was registered with the PROSPERO International Prospective Register of Systematic Reviews (PROSPERO 2022 CRD42022354525). This meta-analysis was not based on the individual participant data, thus ethical approval was not applicable.

A systematic search of studies published between 2000 and 2022 was conducted through PubMed, Cochrane Library, Embase (Elsevier), CINHAL (EBSCOhost), Scopus (Elsevier), and Web of Science: Core Collection (Clarivate Analytics) databases. The initial search strategies were performed: “oxidized phospholipid” OR “oxPLs” OR “oxidized LDL-C” OR “oxidized low-density lipoprotein” OR “oxLDL” OR “low-density lipoprotein receptor-1” OR “LOX-1” OR “sLOX-1” OR “apoA-I” OR “Apolipoprotein A-I” OR “Apolipoproteins E” OR “apolipoprotein E” OR “ApoE” OR “ApoC2” OR “ApoC3” OR “oxHDL” OR “Lipoproteins, LDL” OR “Lipoproteins, HDL” OR “modified lipoprotein” and (“Myocardial Infarction” OR “Stroke” OR “Cerebral” OR “Angina Pectoris” OR “Arteriosclerosis” OR “atherogenes” OR “atherosclerotic” OR “coronary artery disease” OR “Psoriasis”) and (“Patient Outcome Assessment” OR “Risk Assessment” OR “Treatment Outcome”). While we initially planned to include all oxidized lipids in our systematic review, the results of the search strategy were ultimately focused on oxidized low-density lipoprotein as this search term yielded the greatest number of relevant studies. We also considered reference lists and review articles for other potentially relevant citations. The references of retrieved articles were also reviewed to identify any relevant study. Language restriction of English was applied. We used Endnote software (Clarivate Analytics, Philadelphia, PA) for management of the studies.

### Study selection criteria

A 2-step selection process was conducted using Covidence (Covidence, Melbourne, Victoria, Australia) screening software. In the first step, titles and abstracts generated from the search strategy were reviewed by two independent researchers. Studies that did not examine the association between oxidized low-density lipoprotein, chronic inflammatory conditions, and cardiovascular disease measures were excluded. In the second step, studies successfully screened in after the first step were reviewed in full text to confirm if they reported the mean with standard deviation, or median with interquartile range for observational studies.

### Inclusion and exclusion criteria

Inclusion criteria were: observational studies investigating the relationship between oxLDL and CVD in patient populations with chronic inflammatory diseases, including psoriasis, systemic lupus erythematosus, rheumatoid arthritis, and human immunodeficiency virus.

Exclusion criteria were: incorrect study design; literature reviews, discussions, editorials, opinion pieces, and abstracts-only texts; wrong comparators; incorrect setting; wrong patient population; studies that did not report mean and standard deviation or median with interquartile range for observational studies; unavailable full text articles.

### Data extraction and quality assessment

After the 47 available full-text articles were selected, 3 full-text sources were examined for representative data containing effect size (ES) of oxLDL measured by mean and standard deviation or median and interquartile range. For these studies, Covidence software was used to extract the data. The following data were extracted from each included study: first author's name, publication year, number of subjects, participant population, type of publication, patient characteristics (mean or median age in years, percentage of men, baseline body mass index), effect size of oxLDL (represented by mean with standard deviation, median with interquartile range), and study outcomes [defined as incident CVD event, carotid intima-media thickness, or coronary plaque presence as measured by coronary computed tomographic angiography (CCTA)]. To standardize the different measurements and units of oxLDL reported in the studies used in our analysis, we utilized the standardized mean difference with 95% confidence interval to consistently compare oxLDL across studies. Any studies representing results through median with interquartile range (IQR) were converted to mean with standardized mean difference based on methods from Wan et al. (36). All data extractions were completed by two reviewers (EF, HL) and checked by another reviewer (CGH).

### Statistical analyses

The pooled standardized mean difference with its 95% confidence interval (CI) was calculated for oxLDL to account for the different units of oxLDL measurement across all studies. Statistical heterogeneity was identified if the *P* value for Cochran Q was < 0.05 or the *I*^2^ statistics was >50% (37). The Hedges random effects model was chosen if heterogeneity was detected (38). Otherwise, an inverse variance fixed effect model was used. Publication bias was considered if P < 0.05 for the Egger or Begg test along with the funnel plot method (Supplementary Figure 1). All statistical analyses were performed using R Statistical Software (version 4.2.0, R Foundation for Statistical Computing, Vienna, Austria).

## Results

### Study selection

**Figure 1:**
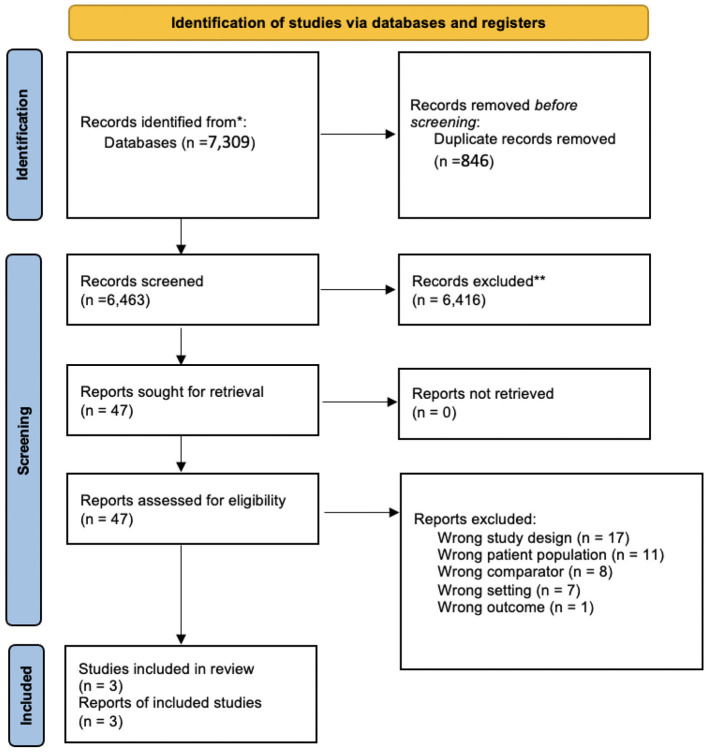
Flow chart of the study selection generated by PRISMA.

The screening and selection process is demonstrated using a flowchart diagram in Figure 1. Initially, a total of 7,309 relevant studies were imported into Covidence with 846 duplicates immediately removed. Of the 6,463 remaining references, 6,416 were excluded in the first step of the selection strategy based on title and abstract screening. Review of the remaining 47 studies in full text form during the second step of the selection strategy yielded 3 final studies (39–41) with 1,060 participants that were included in the meta-analysis. While excluded from the meta-analysis, 4 additional studies from the 47 full-text sources were included in our discussion for their findings on other promising biomarkers of LDL oxidation, including LDL-conjugated dienes, soluble lectin-like oxidized LDL receptor-1 (sLOX-1), oxidized phospholipids (Ox-PLs), and other oxidation-modified lipoproteins (OMLs) (27, 33, 42, 43).

### Study characteristics

The studies included in our meta-analysis are shown in Table 1. The sample size of individual study ranged from 105 to 755 participants. Of the three studies, one was cross-sectional (40) and the rest were cohort studies (39, 41). All the studies were published between 2010 and 2021 and only included participants without known CVD history. The enrolled participants had a mean age range from 38.96 to 51 years. The sex by percent male in the studies ranged from 31.5 to 77%. The baseline body mass index (BMI) ranged from 23.3 to 28.0. All three studies measured oxLDL concentration using an enzyme-linked immunosorbent assay (ELISA) (Mercodia, Uppsala, Sweden). One study reported oxLDL as per the change in oxLDL levels (ΔoxLDL) (39), one study reported oxLDL levels in U/L (40), and one study reported oxLDL levels in mU/L (41). Thus, to account for the different measurements of oxLDL reported in these studies, the pooled standardized mean difference with its 95% confidence interval (CI) was calculated. In order to assess the effect size (ES) of oxLDL and CVD, two studies utilized the odds ratio (OR) (40, 41) and one study used the hazard ratio (HR) (39) (Table 1). All quality scores of the included studies were calculated as >5 according to the Newcastle-Ottawa Scale (NOS) (44). NOS scores of the studies included in the meta-analysis are presented in Table 2.

**Table 1:** Baseline characteristics of studies included in the meta-analysis.

**Reference**	**Design**	**Study Population**	**Age**	**Sex, male%**	**BMI**	**oxLDL mean (SD)**	**oxLDL assay**	**Adjusted variables**		**CVD outcome**	**Effect size (95% CI)**
								**oxLDL exposure**	**Other variables**		
Ajeganova et al. (38)	Cohort (hospital-based)	114 RA patients from the BARFOT trial	50.6 ±11.2	31.5	24.93 ± 4	Case: 56.4 (12.69); Control: 54.2 (15.5)	Mercodia	ΔoxLDL	Age	Occurrence of MI, Angina pectoris, congestive HF, ischemic cerebrovascular event	HR 1.03 [1.0–1.06] 0.035
Parra et al. (39)	Cross-sectional	187 HIV patients at Hospital Universitari de Sant Joan	38.96 ± 0.61	68.8	23.31 ± 0.27	Case: 97.8 (29.22); Control: 76.22 (28.43)	Mercodia	U/L	Age, gender, smoking status, SBP, DBP, glucose, LDL-C, HDL-C, TG, BMI, HIV-1 basal viral load, basal CD4 cell count, lipodystrophy, exposure time to NRTI, NNRTI and (PI) treatments, inflammatory markers, and oxidative markers	Atherosclerosis evaluated by carotid intima-media thickness (CIMT). CVD risk estimated using FRS, low risk (< 10%), moderate (10–20%) and high risk (>20%)	OR 1.026 [1.001–1.05]
Hoffmann et al. (40)	Cohort (community-based)	755 HIV-positive participants from the REPRIEVE study	51 ± 6	77	28.0 ± 6.0	Case: 382.27 (138.09) Control: 340.03 (116.48)	Mercodia	mU/L	ASCVD risk, HIV Parameters (ART duration, CD4, nadir CD4), age, sex, and race, LDL-C level, HTN, and current smoking	Prevalence and composition of CAD measured as coronary plaque on coronary CTA	OR 1.01 [0.90–1.15]

BARFOOT, Better Anti-Rheumatic Pharmaco-Therapy; REPRIEVE, Randomized Trial to Prevent Vascular Events in HIV; SBP, systolic blood pressure; DBP, diastolic blood pressure; LDL-C, low-density lipoprotein cholesterol; HDL-C, high-density lipoprotein cholesterol; TG, triglycerides; BMI, body mass index; HIV-1, human immunodeficiency virus-1; NRTI, nucleoside reverse transcriptase inhibitors; NNRTI, Non-nucleoside reverse transcriptase inhibitors; ASCVD, atherosclerotic cardiovascular disease; ART, anti-retroviral therapy; HTN, hypertension; MI, myocardial infarct; HF, heart failure; FRS, Framingham risk score; CAD, coronary artery disease; CTA, computed tomography angiography.

**Table 2:** Quality assessment of studies included in meta-analysis.

**Reference**	**Is the case definition adequate?**	**Representative ness of the cases**	**Selection of controls**	**Definition of controls**	**Comparability of groups of basis of design or analysis**	**Ascertainment of exposure**	**Ascertainment of both groups with same method**	**Overall NOS scores**
Ajeganova et al. (38)	⋆	⋆	-	⋆	⋆	⋆	⋆	6
Parra et al. (39)	⋆	⋆	-	⋆	⋆⋆	⋆	⋆	7
Hoffmann et al. (40)	⋆	⋆	⋆	⋆	⋆⋆	⋆	⋆	8

NOS, Newcastle-Ottawa Scale.

### Elevated oxLDL significantly associates with CVD in inflamed populations

**Figure 2:**
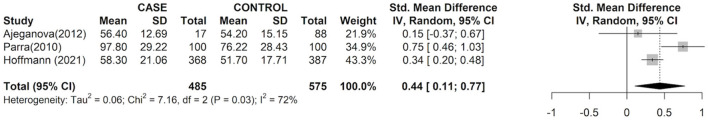
Effect size between circulating oxidized low-density lipoprotein and atherosclerotic cardiovascular disease risk. Forest plots describing effect size (ES), also known as the standard mean difference, with 95% confidence interval (CI) for all included observational studies. Squares represent study-specific estimates; box sizes represent individual study weight; horizontal line represent 95% CIs; diamonds represent the total mean difference with its 95% CI.

The individual studies and pooled meta-analysis results are demonstrated in Figure 2. Of the three studies, two assessed oxLDL levels in 468 participants with HIV disease and associated CVD (defined by carotid intima-media thickness or coronary plaque presence on coronary CTA) vs. 487 participants with HIV disease without CVD and found that increased oxLDL levels were significantly associated with CVD compared to those without CVD (ES for Parra: 0.75 (0.46, 1.03]; ES for Hoffman: 0.34 [0.20, 0.48]). In one study of participants with rheumatoid arthritis, there was no significant association between oxLDL ES and CVD. As shown in Figure 2, the pooled total effect size of elevated oxLDL indicated that participants with chronic inflammatory diseases with associated CVD had a significant increase in oxLDL compared to those without CVD (0.44 [95% CI: 0.11, 0.77]). Cochran Q and *I*^2^ index indicated that there was heterogeneity observed for the marginal analysis. The heterogeneity may be secondary to different methods of measurements and study participants. *P*-value of the Egger's test for funnel plot asymmetry was 0.86, which does not suggest the presence of publication bias.

## Discussion

Chronic inflammatory conditions, such as psoriasis, RA, HIV, and SLE, have increased oxLDL levels, accelerated atherosclerosis, and premature adverse cardiovascular outcomes (9–13, 45). Thus, these pathologies provide suitable human models to study the mechanisms of inflammatory atherosclerosis and associated CVD in humans. In our systematic review with meta-analysis, we aimed to use these diseases to better understand oxLDL as a CV risk biomarker and its relationship with atherosclerotic CVD within the context of chronic inflammation. We found that compared to chronically inflamed subjects without CVD, elevated oxLDL levels were significantly associated with higher CVD presence in patients with chronic inflammatory conditions (ES total: 0.44 [95% CI: 0.11, 0.77]). These results extend the current understanding of the clinical utility of oxLDL as a potential biomarker for CV risk assessment in chronically inflamed populations.

Oxidized LDL is known to have pro-inflammatory and pro-atherogenic properties (46) and can predict increased risk of myocardial infarction (MI) (47–49). Additionally, many studies have demonstrated elevated levels of oxLDL in chronic inflammatory populations. Autoantibodies against oxLDL (auAb-oxLDL) were shown to be elevated in patients with psoriasis compared to matched controls, with 42% of psoriasis patients and 3.3% of control subjects having higher auAb-oxLDL levels than the cut-off point (352 mU/mL) (50). The autoantibody levels were also found to significantly correlate with the Psoriasis Area Severity Index score, a tool used to assess the severity and extent of psoriasis (50). OxLDL also significantly associated with noncalcified coronary burden, a marker of subclinical atherosclerosis, in patients with psoriasis (33). A recent study comparing female lupus patients with and without CVD found that oxLDL was significantly higher in those with CVD (14). However, to our knowledge, this is the first meta-analysis to observe the association between oxLDL and CVD only within chronic inflammatory disease populations. Thus, our aim for this systematic review and meta-analysis was to: (1) to summarize current literature on the relationship between oxLDL and CVD in chronic inflammatory populations, and (2) to provide a standardized representation of measured oxLDL levels across various studies. Currently, no standardized units or reference levels exist for reporting oxLDL measured by different biochemical assays (51–54). Thus, our findings utilized the standardized mean difference to unify the reporting of oxLDL across different studies.

### Pharmacological perspective on oxLDL as a potential therapeutic target

Low-density lipoprotein cholesterol is a prognostic circulating biomarker for stratifying general cardiovascular risk (20, 21). Consequently, lipid-lowering treatments, such as statins and fibrates, are the mainstay treatments of lowering LDL-C levels as well as decreasing triglycerides, increasing HDL-C levels, and reducing hepatic cholesterol biosynthesis (55, 56). However, there is still a need for more specific biomarkers with pathological relevance, especially in chronically inflamed populations, to improve the risk stratification of cardiovascular events (57). OxLDL may be a promising candidate, as increased oxLDL levels are central to atherosclerotic plaque formation and thus may be more causally associated with CVD outcomes than LDL-C (31, 58). Several studies have illustrated the association between elevated circulating oxLDL and adverse CVD outcomes (31, 58, 59). More importantly, Tsimikas et al. demonstrated that high dose atorvastatin reduced total plasma oxidized phospholipids complexed with apolipoprotein B-100 (ApoB-100), the primary protein of the LDL particle, suggesting that statins may partly exert protective cardiovascular effects through mobilization of pro-inflammatory oxidation species from atherosclerotic lesions (60). Further, the “Standard vs. high-dose therApy with Rosuvastatin for lipiD lowering” (SARD) randomized clinical trial found that high dose rosuvastatin significantly reduced levels of oxLDL when compared to low dose rosuvastatin (61). In the setting of HIV, several studies showed that statin therapy reduced noncalcified coronary plaque volume, total plaque volume, and positively remodeled plaque in patients with HIV (62, 63) (Table 1). Thus, using current statin therapy to treat elevated oxLDL levels in addition to LDL-C may provide increased benefits in potentially reducing the risk of adverse CVD events in such populations.

While statins are the mainstay lipid-lowering therapy, many people are statin intolerant or cannot achieve goal LDL-C levels on statin therapy alone and thus require alternative therapy. Injectable lipid-lowering therapy currently used for inherited hypercholesterolemia and high-risk CV patients have demonstrated great benefit for such patients (64). Proprotein convertase subtilisin/kexin type 9 (PCSK9) inhibitors are a type of injectable lipid-lowering therapy targeting PCSK9, a protease enzyme produced in hepatocytes involved in LDL binding, internalization, and degradation (65, 66). Interestingly, PCSK9 is also implicated in the oxLDL-induced inflammatory pathway. In adult rat ventricular cardiomyocytes, oxLDL significantly impaired contractile function *via* induction of PCSK9 (67). Tang et al. found that PCSK9 small interfering RNA suppressed oxLDL-induced inflammatory response in THP-1-derived macrophages (68). Thus, PCSK9 inhibitors offer a novel therapeutic opportunity in targeting oxLDL-related atherosclerotic outcomes (67). Evolocumab is a PCSK9 inhibitor that when added to maximally tolerated statin therapy was found to reduce the risk of cardiovascular outcomes in patients with atherosclerotic CVD, while data from the “ODYSSEY OUTCOMES” trial demonstrated decreased risk of recurrent ischemic cardiovascular events in patients with previous acute coronary syndrome treated with alirocumab in addition to high-intensity statins (65, 69, 70). These findings illustrate the importance of discovering additional treatment modalities for patients at high risk for CVD complications.

In addition to oxLDL as a candidate biomarker for CVD, there is a growing body of literature showing the potential role of anti-oxLDL antibodies and other oxLDL-related moieties for CVD risk stratification and promising therapeutic targets (71). Autoantibodies to oxidation-specific epitopes on LDL, such as MDA-modified LDL (MDA-LDL), are found in atherosclerotic lesions of humans and animals (72, 73) and there is significant research on the clinical correlates of these antibodies (74–76). Karvonen et al. demonstrated that IgM autoantibodies to MDA-LDL epitope had an inverse association with carotid atherosclerosis in a population cohort study of 1,022 middle-aged men and women (77). Soluble lectin-like oxLDL receptor 1 (sLOX-1) is an inflammation-induced receptor for oxLDL that has been shown to induce myocardial ischemia through unstable atherosclerotic plaque formation, suggesting an important role of LOX-1 in the pathogenesis of oxLDL-related CVD (78, 79). Interestingly, increased sLOX-1 levels have been associated with systemic inflammatory diseases. sLOX-1 levels were higher in patients with RA with positive rheumatoid factor and anti-citrullinated protein antibody serology than those without, and continued to remain at high levels in non-remission patients compared to those in remission irrespective of treatment, highlighting the potential utility of sLOX-1 as a biomarker for disease activity and remission in RA (80). SLE patients had two-fold higher levels of sLOX-1, which positively associated with high-sensitivity CRP levels, oxLDL, proinflammatory HDL, and impaired HDL efflux, instead of traditional risk factors and SLE disease activity (79). Further, the authors found that SLE patients with higher sLOX-1 levels were younger than those with low levels, which is concerning given that SLE patients are at greater risk of CVD and is particularly evident in the younger female SLE population, as 54% of cardiac events that occur in female SLE patients are under the age of 44 (81). Thus, elevated sLOX-1 levels may serve as an useful biomarker of increased CVD risk, and sLOX-1 inhibition may be a therapeutic opportunity for decreasing atherosclerosis in these patients. Additionally, genetic modulation has become a promising therapeutic approach for oxLDL treatment. For instance, overexpression of the long non-coding RNA LINC00452 has been shown to reverse oxLDL injury in human umbilical vein endothelial cells (HUVECS) by regulating the miR-194-5p/IGF1R axis (82). Additionally, miR-214-3p in HUVECS regulates oxLDL-initiated macrophage autophagy, thus suggesting a potential therapeutic role for miRNAs in atherosclerosis (83). Further studies are necessary to elucidate more therapeutic targets aimed at the function and quantity of oxLDL in the pathogenesis of cardiovascular disease.

Several articles excluded based on our exclusion criteria for the meta-analysis were deemed important to include here for their discussion of other oxLDL-related potential biomarkers for optimization of CVD risk stratification in inflamed populations. In a multicenter observational study, Nyyssönen et al. found that increased LDL-conjugated diene concentrations, identified as one of the first stages of LDL oxidation and subclinical atherosclerosis, exhibited a positive relationship with increased CIMT in high-risk subjects presenting with at least three vascular risk factors (VRF) (27). Oxidation-specific biomarkers primarily oxidized phospholipids (Ox-PLs) on apolipoprotein B-100-containing lipoproteins (oxPL/ApoB-100), have been demonstrated as essential in identifying the risk of peripheral artery disease (43). Other studies have focused on the uptake pathway of oxLDL through soluble lectin-like oxidized LDL receptor-1 (sLOX-1) to better understand atherosclerosis. Dey et al. showed that in patients with psoriasis, sLOX-1 associated with imaging markers of subclinical atherosclerosis and increased psoriasis severity (42). Moreover, patients with psoriasis had decrease in plasma levels of oxidation-modified lipoproteins, including oxLDL under specific biologic treatment (33, 42).

### Other potential oxLDL-related biomarkers of CVD and atherosclerosis

Other LDL-related lipoproteins, including sdLDL and Lp (a), are prone to oxidation and associated with elevated cardiovascular risk (84–86). Because of their physical and compositional characteristics, they have higher affinity for extracellular matrix, reduced binding to LDL receptor, and increased residence time in the circulation compared to large (buoyant) LDL particles (23, 87, 88). In patients with psoriatic arthritis, sdLDL concentration was increased independently of the presence of metabolic syndrome, suggesting a potential mediation by sdLDL of atherosclerosis development in psoriatic arthritis (89). Furthermore, a study comparing HIV-positive with HIV-negative participants found increased sdLDL levels in those with HIV (63). Both HIV infection and combination antiretroviral therapy are thought to induce endothelial dysfunction through endothelial cell activation, oxidative stress, and inflammation that leads to increased cardiovascular disease in these patients (90). Indeed, in a study by Post et al., the authors found that suboptimal HIV RNA suppression and combined antiretroviral therapy adherence were the main determinants of coronary artery stenosis progression during a median follow-up of 4.5 years (91). Lp (a) is also a candidate biomarker and has been demonstrated to predict CIMT in HIV-positive females (92). While these findings are promising, meta-analyses investigating the role of oxidized lipids within systemically inflamed populations are lacking and thus future studies will continuously be necessary to further elucidate these relationships (93, 94).

Our meta-analysis had several limitations. Firstly, the causal association between oxLDL and CVD outcomes in our populations of interest could not be defined because of the cohort or cross-sectional nature of the included studies. Another limitation is that studies using other techniques to estimate CVD outcomes were not included in this meta-analysis. As observational studies show more heterogeneity than randomized control trials and several of the included studies were observational studies, this factor must also be considered given that heterogeneity interferes with the detection of publication bias (95, 96). The heterogeneity sources may correlate with study design, participant ages, and whether patients have atherosclerotic risk factors. While oxLDL is a promising biomarker for CVD risk stratification, oxLDL is not yet used in the clinic as a diagnostic tool for CVD. Finally, we were unable to determine the effects of populational characteristics or pharmacologic therapy on the progression of CVD outcomes in relation to oxLDL in patients with chronic inflammatory diseases.

## Conclusion

Our systematic review and meta-analysis demonstrate that patients with chronic inflammatory diseases, particularly RA and HIV, have significantly higher levels of circulating oxLDL as measured by effect size in relation to increased cardiovascular risk. Thus, oxLDL may offer insight into optimizing CVD risk stratification in chronically inflamed populations. We also discussed additional atherogenic lipoprotein parameters associated with oxLDL that offer a more nuanced understanding of lipoprotein modifications linked with CVD in the setting of inflammation. Larger meta-analysis and future mechanistic studies are necessary to further elucidate the relationship between oxidized lipoproteins and cardiovascular disease in patients with long-standing inflammatory conditions.

## Data availability statement

The original contributions presented in the study are publicly available. This data can be found here: https://www.crd.york.ac.uk/prospero/#searchadvanced, accession number: CRD42022354525.

## Author contributions

CH, EF, HL, and AS were involved in designing the concept of the review and oversight and in the literature search, summation of the literature, and revisions of the manuscript. CH, EF, HL, PP, NM, and AS drafted the manuscript. All authors read, reviewed, and approved the final manuscript.
