# Supplementary material for: Oxidized low-density lipoprotein associates with cardiovascular disease by a vicious cycle of atherosclerosis and inflammation: A systematic review and meta-analysis
Source: Front Cardiovasc Med. 2023 Jan 16;9:1023651. doi: 10.3389/fcvm.2022.1023651 (PMC9885196; doi:10.3389/fcvm.2022.1023651)
Supplement: Supplementary file 1 [file Image_1.PDF]

SUPPLEMENT

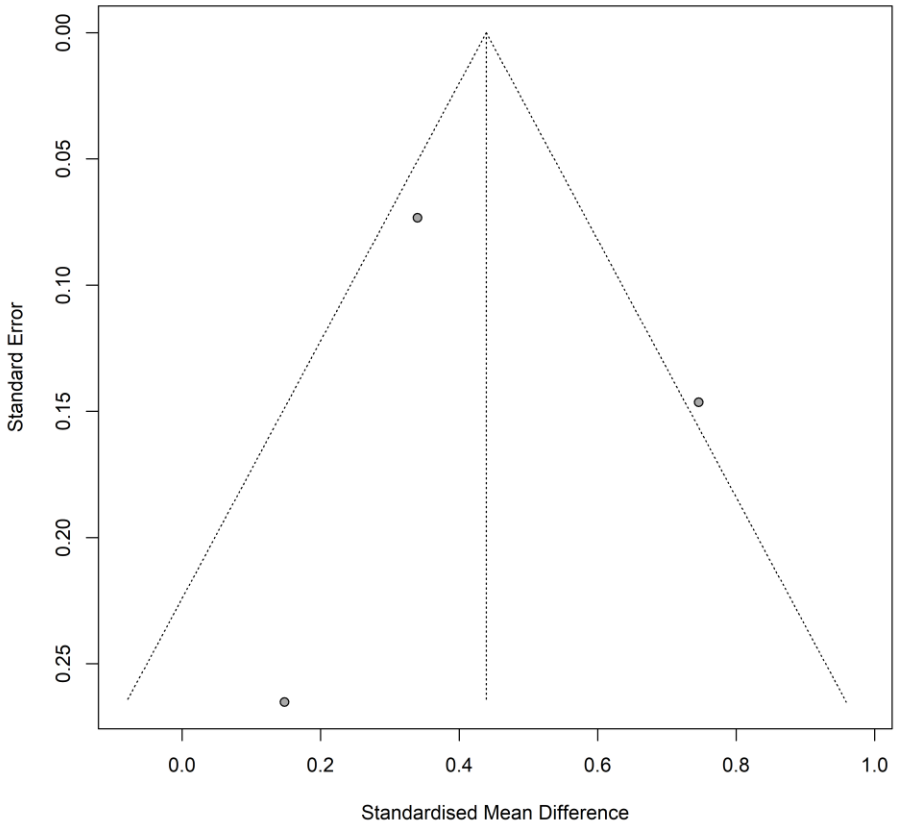

Supplemental Figure 1: Funnel plot was used to assess publication bias of the study. P-value=0.86 which does not suggest the presence of publication bias.
